# Questionable prospective effect of perfectionism on depression: reanalysis of a meta-analytic cross-lagged panel analysis

**DOI:** 10.3389/fpsyg.2025.1378482

**Published:** 2025-07-02

**Authors:** Kimmo Sorjonen, Bo Melin

**Affiliations:** Department of Clinical Neuroscience, Karolinska Institutet, Stockholm, Sweden

**Keywords:** depressive symptoms, meta-analysis, overinterpretation of findings, perfectionism, reanalysis, spurious cross-lagged effects, vulnerability model

## Abstract

**Background:**

A recent meta-analysis claimed increasing prospective effects between perfectionism and depressive symptoms. However, the effects were estimated while adjusting for a prior measurement of the outcome variable and it is known that such adjusted cross-lagged effects may be spurious due to correlations with residuals and regression to the mean.

**Method:**

We reanalyzed the same meta-analytic data as in the challenged study with alternative regression models.

**Results:**

Alternative models indicated contradictory increasing and decreasing prospective effects of perfectionism on depressive symptoms.

**Conclusion:**

The present divergent findings suggested that the prospective effect of perfectionism on depressive symptoms may have been spurious. Consequently, the conclusions in the challenged meta-analysis, and the vulnerability model of perfectionism and depressive symptoms in general, can be called into question. It is important for researchers to be aware of the limitations of cross-lagged panel analyses, and of correlations in general, in order not to overinterpret findings.

## Introduction

Depression is a leading cause of disability worldwide (James et al., 2018). Consequently, it is vital for researchers to identify causes of depression. Perfectionistic concerns and strivings are two dimensions of perfectionism. Perfectionistic concerns are socially prescribed and encompass beliefs that others require perfection of the self, doubts that one can live up to the standards, and self-criticism when one allegedly fails others' expectations. Perfectionistic strivings, on the other hand, are self-oriented and include high personal standards and lofty goals (Frost et al., 1993; Smith et al., 2021). Studies have shown a positive association between depressive symptoms and perfectionism, where associations with perfectionistic concerns tend to be stronger than associations with perfectionistic strivings (Limburg et al., 2017).

According to the vulnerability model, some personality traits, including perfectionism, cause people to think, feel, and behave in ways that increase the risk for depressive symptoms (Hewitt et al., 1996; Graham et al., 2010; Sherry et al., 2013, 2014). Contrarily, according to the complication model, depressive symptoms may cause changes in personality, including perfectionism (Bagby et al., 2008). The reciprocal relations model incorporates both the vulnerability and complication models and suggests dynamic and bidirectional effects between perfectionism and depressive symptoms (McGrath et al., 2012).

Smith et al. (2021) conducted a meta-analytic cross-lagged panel analysis and found a significant positive effect of initial perfectionism on subsequent depression when adjusting for initial depression. For perfectionistic concerns, but not for perfectionistic strivings, Smith et al. also found a reversed effect of initial depression on subsequent perfectionism when adjusting for initial perfectionism. Smith et al. concluded that individuals with elevated perfectionistic concerns appear to be trapped in a vicious loop with ever-increasing levels of depression and perfectionistic concerns.

However, it is known that cross-lagged effects while adjusting for a prior measurement of the outcome variable may be spurious due to correlations with residuals and regression to the mean (Glymour et al., 2005; Eriksson and Häggström, 2014; Castro-Schilo and Grimm, 2018; Sorjonen et al., 2019). As an example, picture individuals with the same initial depression score but with different initial perfectionism scores. Due to the positive association between perfectionism and depression, we should suspect that individuals with a high initial perfectionism score have received a low initial depression score compared with their true degree of depression, i.e., they have received a negative residual in the measurement of depression. Contrarily, individuals with a low initial perfectionism score have probably received a high initial depression score compared with their true degree of depression, i.e. they have received a positive residual in the measurement of depression. However, residuals tend to regress toward a mean value of zero between measurements. Consequently, we should expect a more positive, but spurious, change in the depression score to a subsequent measurement for those with a high initial perfectionism score compared with those with the same initial depression score but with a lower initial perfectionism score. Furthermore, as regression toward the mean is independent of the direction of time, if the effect is spurious, we should also expect a positive effect of initial perfectionism on the initial depression score when adjusting for the subsequent depression score.

**Figure 1:**
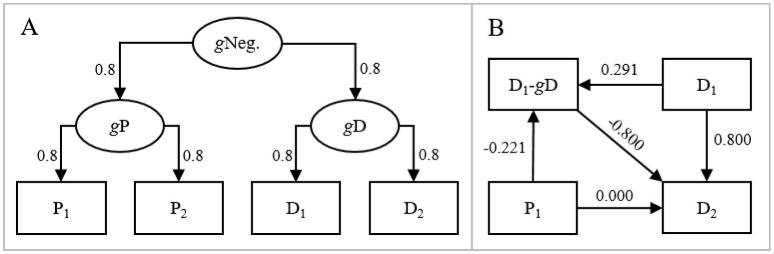
**(A)** Hypothetical data generating model, where general levels of perfectionism and depression are affected by general negativity and where measures of perfectionism and depression at two occasions, in their turn, are affected by general perfectionism and depression, respectively. The lack of direct effects between perfectionism and depression should be noted. **(B)** Path model with standardized regression effects between P_1_, D_1_, D_2_, and the D_1_-*g*D residual if data are generated as in **(A)**.

To elaborate and exemplify, imagine that data are generated as in Figure 1A. Here, individuals' general/true levels of perfectionism (*g*P) and depression (*g*D) are affected by their general level of negativity (*g*Neg.) but have no direct effects on each other. Perfectionism and depression measured at two occasions (P_1_, P_2_, D_1_, and D_2_, respectively) are affected by general levels but have, again, no direct effects on each other. We generated data (*N* = 10,000) with the model in Figure 1A. Although perfectionism and depression had no direct effects on each other, P_1_ had a positive effect on D_2_ when adjusting for D_1_ (β = 0.177, *p* < 0.001, this effect corresponds to −0.221 × −0.800 in Figure 1B). However, if adjusting for the D_1_-*g*D difference/residual in addition to D_1_, P_1_ had no effect on D_2_ (β = 0.000, *p* = 1, Figure 1B). This means that the effect of P_1_ on D_2_ when adjusting for D_1_ was fully accounted for by a negative association between P_1_ and the D_1_-*g*D residual in combination with a negative association between the D_1_-*g*D residual and D_2_.

It has been proposed that one of the problems with cross-lagged panel models (CLPM) is an inability to separate within-individual effects from between-individual differences (Hamaker et al., 2015). The random-intercept cross-lagged panel model (RI-CLPM) is an extension of the traditional CLPM, where longitudinal scores are regressed on stable trait-like latent variables. Then, autoregressive and cross-lagged effects are estimated between within-individual residuals not accounted for by the stable trait-like levels (Hamaker et al., 2015; Mulder and Hamaker, 2021). It has been proposed that such within-individual effects are better estimates of causality than between-individual differences in CLPM (Usami et al., 2019). However, RI-CLPM is not able to account for time-varying confounders (Rohrer and Murayama, 2023; Murayama and Gfrörer, 2024). This means that RI-CLPM is susceptible to similar spurious findings as CLPM and do, consequently, not allow strong causal inference. We have suggested that causal conclusions based on findings by the RI-CLPM can be scrutinized, similarly as causal conclusions based on the CLPM, by fitting alternative models to data (Sorjonen et al., 2023d, 2025b; Sorjonen and Melin, 2025). It should be noted that the meta-analytic data analyzed by Smith et al., and reanalyzed by us, included two waves of measurement and could, consequently, not be analyzed with the RI-CLPM, which requires data from at least three waves of measurement.

The objective of the present study was to reanalyze the meta-analytic associations used by Smith et al. (2021) in order to evaluate if the prospective effects between perfectionism and depression may have been spurious due to correlations with residuals and regression to the mean rather than, as suggested by Smith et al., truly increasing.

## Method

See Smith et al. (2021) for a more comprehensive description of selection of studies, sample characteristics, etc. In short, Smith et al. extracted autoregressive, concurrent, and cross-lagged zero-order correlations between perfectionism and depressive symptoms measured at two occasions from 67 studies with data from 77 samples [total *N* = 20,583, mean age = 25.8 (*SD* = 11.6), average percentage female participants = 65.4% (*SD* = 25.4)].

**Figure 2:**
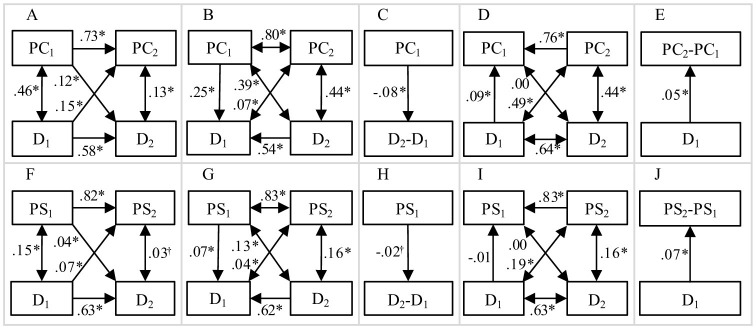
Standardized associations between depressive symptoms **(D)** and perfectionistic concerns (PC, **A–E**) and perfectionistic strivings (PS, **F–J**) in: **(A, F)** A traditional cross-lagged panel analysis; **(B, G)** A reversed cross-lagged panel analysis where initial perfectionism predicted initial depressive symptoms while adjusting for subsequent depressive symptoms; **(C, H)** A model where initial perfectionism predicted subsequent change in depressive symptoms; **(D, I)** A reversed cross-lagged panel analysis where initial depressive symptoms predicted initial perfectionism while adjusting for subsequent perfectionism; **(E, J)** A model where initial depressive symptoms predicted subsequent change in perfectionism;^†^*p* < 0.05; *p < 0.001

In turn, we extracted the 2 × 6 meta-analytic zero-order correlations between depressive symptoms and perfectionistic concerns and strivings, respectively, measured at two occasions, from Smith et al. We simulated two datasets with these correlations between variables and with sample sizes *N* = 16,131 and *N* = 11,494 for perfectionistic concerns and strivings, respectively. These sample sizes corresponded to total sample sizes in Smith et al. We fitted various models (Figure 2) to data in order to discriminate between truly increasing and spurious prospective effects:

(1) A traditional cross-lagged panel model, where initial perfectionism predicted subsequent depressive symptoms while adjusting for initial depressive symptoms, and vice versa (Figures 2A, F). Here, both a hypothesis of true increasing and a hypothesis of spurious prospective effects predicted positive effects (Table 1, rows 1 and 4);
(2) A reversed cross-lagged panel model, where initial perfectionism predicted initial depressive symptoms while adjusting for subsequent depressive symptoms (Figures 2B, G), and vice versa (Figures 2D, I). Here, a hypothesis of true increasing prospective effects predicted negative effects. This would mean that among individuals with the same subsequent degree of depressive symptoms (e.g., the mean standardized value of zero), those with high initial perfectionism had tended to have lower initial degree of depressive symptoms (e.g., −0.5) and those with low initial perfectionism had tended to have higher initial degree of depressive symptoms (e.g., 0.5). Consequently, those with high initial perfectionism had experienced a more positive change in depressive symptoms between measurements [0–(−0.5) = 0.5] compared with those with the same subsequent degree of depressive symptoms but with low initial perfectionism (0–0.5 = −0.5). Similarly, a negative effect would mean that individuals with high initial degree of depressive symptoms had experienced a larger subsequent increase in perfectionism compared with individuals with the same subsequent perfectionism but with lower initial degree of depressive symptoms. Contrarily, as regression to the mean is independent of the direction of time, a hypothesis of spuriousness predicted a positive effect of initial perfectionism on initial degree of depressive symptoms when adjusting for subsequent degree of depressive symptoms, and vice versa (Table 1, rows 2 and 5). These reversed models were in line with proposals that time-reversed analyses can be used to detect statistical artifacts (Campbell and Kenny, 1999; Haufe et al., 2013);
(3) A hypothesis of truly increasing effects predicted a positive effect of initial perfectionism on the subsequent degree of depressive symptoms—initial degree of depressive symptoms difference (Figures 2C, H), and vice versa (Figures 2E, J). Equation 1 (Guilford, 1965) gives the expected effect of initial perfectionism (X1) on the subsequent degree of depressive symptoms—initial degree of depressive symptoms difference (Y2-Y1), and vice versa. A hypothesis of spuriousness predicted this effect to be either close to zero (if the concurrent, *r*_*X*1, *Y*1_, and cross-lagged, *r*_*X*1, *Y*2_, correlations were approximately equally strong) or negative (if the concurrent correlations were stronger than the cross-lagged correlations) (Table 1, rows 3 and 6).

(1) $$\begin{array}{l} E\left(\beta_{X1,Y2-Y1}\right)=\frac{r_{X1,Y2}-r_{X1,Y1}}{\sqrt{2\left(1-r_{Y1,Y2}\right)}} \end{array}$$

**Table 1:** Predicted sign (positive or negative) of effects between perfectionism and depressive symptoms according to a hypothesis of true increasing reciprocal effects and a hypothesis of spuriousness.

**Effect^a^**	**True**	**Spurious**
1. β(p1,d2.d1)	Positive	Positive
2. β(p1,d1.d2)	Negative	Positive
3. β(p1,d2-d1)	Positive	Zero or negative
4. β(d1,p2.p1)	Positive	Positive
5. β(d1,p1.p2)	Negative	Positive
6. β(d1,p2-p1)	Positive	Zero or negative

p, perfectionism; d, depression; 1, time 1; 2, time 2.

a The variables are given in the order predictor, outcome, and covariate.

Simulations and analyses were conducted with R 4.1.3 statistical software (R Core Team, 2025) employing the MASS (Venables and Ripley, 2002) and lavaan (Rosseel, 2012) packages. Analytic script, which also generates the simulated data, is available at the Open Science Framework at https://osf.io/6mr9b/.

## Results

**Figure 3:**
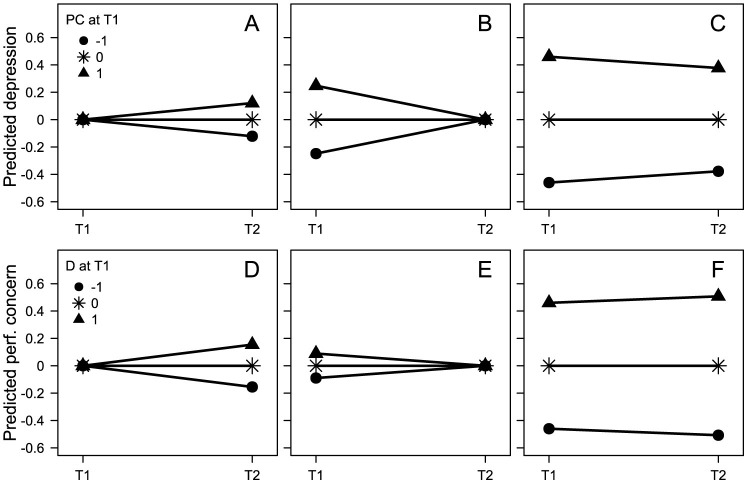
Predicted initial and subsequent depressive symptoms **(A–C)** and perfectionistic concerns **(D–F)**. Separately for those with high (*Z* = 1), average, and low (*Z* = −1) initial perfectionistic concerns **(A–C)** and initial depressive symptoms **(D–F)**, respectively. Separately for situations when conditioning on average initial depressive symptoms **(A)**, average initial perfectionistic concerns **(D)**, average subsequent depressive symptoms **(B)**, average subsequent perfectionistic concerns **(E)**, and when not conditioning on the outcome variable **(C,F)**. The values for this figure come from the models presented in Figure 2, i.e., *b* = 0.12 **(A)** and *b* = 0.15 **(D)** from Figure 2A, *b* = 0.25 **(B)** from Figure 2B, *b* = −0.08 **(C)** from Figure 2C, *b* = 0.09 **(E)** from Figure 2D, *b* = 0.05 **(F)** from Figure 2E. Figures with perfectionistic strivings instead of, as here, perfectionistic concerns, would have looked very similar to the present figures.

Correlations and standardized regression effects in the different models are presented in Figure 2. Initial perfectionism had a positive effect on subsequent depressive symptoms while adjusting for initial depressive symptoms, and vice versa (Figures 2A, F). This suggested, as already shown by Smith et al. (2021), a more positive subsequent change (i.e., a larger increase or a smaller decrease) in depressive symptoms for those with high, compared with low, initial perfectionism but with the same initial degree of depressive symptoms (e.g., D_2_ = 0.12 × PC_1_ + 0.58 × D_1_ + e, Figure 3A), and vice versa (e.g., PC_2_ = 0.15 × D_1_ + 0.73 × PC_1_ + e, Figure 3D).

However, the effects of initial perfectionism on initial depressive symptoms while adjusting for subsequent depressive symptoms (Figures 2B, G), and vice versa (Figure 2D), were also positive (except for the effect of initial depressive symptoms on initial perfectionistic strivings, Figure 2I). This means that among individuals with the same subsequent degree of depressive symptoms, those with high initial perfectionism had tended to have higher initial degree of depressive symptoms and, consequently, to have experienced a more negative subsequent change (i.e., a larger decrease or a smaller increase) in depressive symptoms compared with those with the same subsequent degree of depressive symptoms but with lower initial perfectionism (e.g., D_1_ = 0.25 × PC_1_ + 0.54 × D_2_ + e, Figure 3B), and vice versa (e.g., PC_1_ = 0.09 × D_1_ + 0.76 × PC_2_ + e, Figure 3E).

Moreover, the negative crude effect of initial perfectionism on the subsequent depressive symptoms—initial depressive symptoms difference (Figures 2C, H) suggested a more negative subsequent change (i.e., a larger decrease or a smaller increase) in depressive symptoms for those with high, compared with low, initial perfectionism (e.g., D_2_-D_1_ = −0.08 × PC_1_ + e, Figure 3C). Contrarily, a positive effect of initial depressive symptoms on the subsequent perfectionism—initial perfectionism difference (Figures 2E, J) suggested a more positive subsequent change (i.e., a larger increase or a smaller decrease) in perfectionism for those with high, compared with low, initial degree of depressive symptoms (e.g., PC_2_-PC_1_ = 0.05 × D_1_ + e, Figure 3F).

In summary, effects of perfectionism on depressive symptoms agreed better with a hypothesis of spuriousness than with a hypothesis of true reciprocal effects (compare effects in Figure 2 with predictions in Table 1). Effects of depressive symptoms on perfectionism were more ambiguous.

## Discussion

As already shown by Smith et al. (2021), in the present reanalyses we found a meta-analytic positive effect of initial perfectionism on subsequent depressive symptoms while adjusting for initial depressive symptoms, and vice versa. This could be seen to indicate, as suggested by Smith et al., that perfectionism and depressive symptoms had reciprocal increasing effects on each other. However, we also found a positive effect of initial perfectionism on initial depressive symptoms while adjusting for subsequent depressive symptoms, and a negative effect of initial perfectionism on the subsequent depressive symptoms—initial depressive symptoms difference. These findings suggested, contrarily, a negative effect of perfectionism on subsequent change in depressive symptoms. These contradictory findings indicated that Smith al.s' meta-analytically estimated prospective effect of perfectionism on depressive symptoms may have been spurious due to correlations with residuals and regression to the mean rather than truly increasing. Consequently, the present findings call the vulnerability model of perfectionism and depressive symptoms into question.

Effects of depressive symptoms on perfectionism were more ambiguous. A positive effect of initial depressive symptoms on initial perfectionistic concerns while adjusting for subsequent perfectionistic concerns agreed with a hypothesis of spuriousness. On the other hand, positive effects of initial depressive symptoms on the subsequent perfectionism (both concerns and strivings)—initial perfectionism differences agreed with a hypothesis of a truly increasing effect, i.e., with the complication model. This positive effect was due to a stronger positive meta-analytic correlation between initial depressive symptoms and subsequent perfectionism (*r* = 0.49 and *r* = 0.19 for perfectionistic concerns and strivings, respectively) compared with initial perfectionism (*r* = 0.46 and *r* = 0.15 for perfectionistic concerns and strivings, respectively). Such a temporal strengthening of the association is what can be expected if depressive symptoms had a truly increasing prospective effect on perfectionism. However, as always, caution is advised when/if inferring causality from correlational data.

We have conducted several reanalyses of meta-analyses employing cross-lagged panel analyses and found that most effects may have been spurious, possibly due to correlations with residuals and regression to the mean, rather than truly increasing or decreasing (Sorjonen et al., 2022, 2023a,b,c, 2024, 2025a; Sorjonen and Melin, 2023, 2024a,b). A recurring message in these studies is that cross-lagged effects while adjusting for a prior measurement of the outcome variable usually do not prove anything over and above a cross-sectional association combined with less than perfect reliability in measurements. This limitation of cross-lagged effects, meta-analytically estimated or not, is important for researchers to bear in mind in order not to overinterpret findings, something that appears to have happened to Smith et al. (2021). The continued output of studies with uncritical use of cross-lagged panel analyses indicates that this knowledge, although far from new, is largely missing in the research community. Hence, the limitation of cross-lagged panel analyses is worth repeating. We recommend researchers to employ analyses with a reversed treatment of time, as we have done here, in order to discriminate between true (or, more correctly, “not yet disproven”) and spurious prospective effects.

## Limitations

The present reanalyses suffered from some of the same limitations as the challenged meta-analysis by Smith et al. (2021). For example, 79.4% of the included samples were American, British, or Canadian. Consequently, it remains an open question if the present main finding, that prospective effects of perfectionism on depressive symptoms appear to be spurious due to correlations with residuals and regression to the mean, is generalizable to other cultural contexts.

Measures of perfectionism and depressive symptoms in the studies included in the meta-analysis by Smith et al., and consequently in the present reanalyses, might not always have been optimal. Furthermore, in the present reanalyses we did not consider possible moderating effects of the sex and age composition of the samples, time lag between measurements, etc. However, it is important to note that such factors were constant across the analyzed models and could not, consequently, explain why some models indicated increasing and others decreasing effects between perfectionism and depressive symptoms.

We do not claim to have proven, once and for all, that perfectionism and depressive symptoms have no genuine prospective effects on each other. Correlations in observational (i.e., non-experimental) data can probably never be used to prove causality nor lack thereof. We do claim, however, that a conclusion of spuriousness agrees better with the divergent findings of both increasing and decreasing effects compared with a conclusion of causality. The present findings suggest that the meta-analytic data analyzed by Smith et al. (2021), and reanalyzed by us here, may have been generated without any direct effects between perfectionism and symptoms of depression, e.g., as in the model in Figure 1A.

## Conclusions

The present reanalyses found that a meta-analytic prospective effect of perfectionism on depressive symptoms may have been spurious, possibly due to correlations with residuals and regression to the mean, rather than truly increasing. Hence, the conclusion by Smith et al. (2021), that individuals with elevated perfectionistic concerns appear to be trapped in a vicious loop with ever-increasing levels of depression and perfectionistic concerns, can be called into question. Furthermore, the present findings challenge the vulnerability model which claims an increased risk for depression for those with high levels of perfectionism. It is important for researchers to be aware of the limitations of cross-lagged panel analyses, and of correlations in general, in order not to overinterpret findings.

## Data Availability

Publicly available datasets were analyzed in this study. This data can be found at: Analytic script, which also generates the simulated data, is available at the Open Science Framework at https://osf.io/6mr9b/.
